# In-Line Monitoring of Downstream Purification Processes for VSV Based SARS-CoV-2 Vaccine Using a Novel Technique

**DOI:** 10.3390/biotech10040025

**Published:** 2021-11-03

**Authors:** Arik Makovitzki, Avital Jayson, Ziv Oren, Elad Lerer, Yaron Kafri, Eyal Dor, Lilach Cherry, Hanan Tzadok, Lilach Levin, Ophir Hazan, Irit Simon, Arnon Tal, Meni Girshengorn, Osnat Rosen

**Affiliations:** Department of Biotechnology, Israel Institute for Biological Research, Ness Ziona 7410001, Israel; arikm@iibr.gov.il (A.M.); AvitalJayson@iibr.gov.il (A.J.); zivo@iibr.gov.il (Z.O.); eladl@iibr.gov.il (E.L.); yaronk@iibr.gov.il (Y.K.); eyalo@iibr.gov.il (E.D.); lilachc@iibr.gov.il (L.C.); hanant@iibr.gov.il (H.T.); lilachl@iibr.gov.il (L.L.); ophirh@iibr.gov.il (O.H.); irits@iibr.gov.il (I.S.); arnont@iibr.gov.il (A.T.); menig@iibr.gov.il (M.G.)

**Keywords:** Cobas analyzer, metabolites, nutrients, lactate dehydrogenase (LDH), host cell proteins (HCP), downstream purification process (DSP)

## Abstract

The COVID-19 pandemic caused by Severe Acute Respiratory Syndrome Coronavirus 2 (SARS-CoV-2) increases the need for a rapid development of efficient vaccines. Among other vaccines in clinical trials, a recombinant VSV-∆G-spike vaccine was developed by the Israel Institute for Biological Research (IIBR) and is being evaluated. The development of an efficient downstream purification process (DSP) enables the vaccine to be advanced to clinical trials. The DSP must eliminate impurities, either process- or product-related, to yield a sufficient product with high purity, potency and quality. To acquire critical information on process restrictions and qualities, the application of in-line monitoring is vital and should significantly impact the process yield, product quality and economy of the entire process. Here, we describe an in-line monitoring technique that was applied in the DSP of the VSV-∆G-spike vaccine. The technique is based on determining the concentrations of metabolites, nutrients and a host cell protein using the automatic chemistry analyzer, Cobas Integra 400 Plus. The analysis revealed critical information on process parameters and significantly impacted purification processes. The technique is rapid, easy and efficient. Adopting this technique during the purification process improves the process yield and the product quality and enhances the economy of the entire downstream process for biotechnology and bio pharmaceutical products.

## 1. Introduction

Coronavirus disease (COVID-19) is an infectious disease caused by Severe Acute Respiratory Syndrome Coronavirus 2 (SARS-CoV-2) and mainly comprises symptoms in the lungs, causing cough and fever [1]. The disease has spread worldwide, leading to an ongoing pandemic. The best way to limit the virus transmission while permitting the normal conduct of business and social life is to induce a rapid onset of immune protection by administering a vaccine against COVID-19.

Different vaccine candidates have already been approved around the world for general or emergency use. As of November 2021, more than 300 vaccines are in clinical and pre-clinical trials, all targeting the spike protein of severe acute respiratory syndrome coronavirus type 2 (SARS-CoV-2). Most vaccines are based on either viral vectors or mRNA, all of which encode the spike protein (or a fragment of it). Other vaccines are based on the whole virus (live-attenuated or inactivated), DNA or recombinant protein subunits [2]. One of the vaccines being tested in clinical trials is being developed by the IIBR [3,4]. This vaccine is based on the vesicular stomatitis virus (VSV), in which the VSV-G protein has been replaced with the SARS-CoV-2 spike protein (rVSV-∆G-spike), creating a recombinant replicating virus [3,4,5]. The rVSV-∆G-spike is produced in Vero cells, grown on Fibra-Cel bioreactors [6]. The virus is released into the culture medium and, at the end of the production process, the medium is collected.

To bring the rVSV-∆G-spike to clinical trials, a downstream process (DSP) that will rescue the virus from the medium and is suitable for scale-up is required [7,8]. The ultimate goal of the DSP is to obtain a sufficient product with high purity, potency, safety and quality to meet the stringent guidelines of the regulatory authorities [8]. The DSP must eliminate impurities, either process-related (e.g., DNase, nutrients, extractables and leachables) or product-related (e.g., host cell proteins (HCP), metabolites and host cell DNA (hc-DNA)). The DSP that was developed by the IIBR for the rVSV-∆G-spike vaccine is a multistep process that comprised endonuclease digestion, depth and membrane filter clarification, chromatographic purification and ultrafiltration [9,10].

One of the main challenges in DSP development is monitoring the impurity removal in each step of the process. Monitoring could have a very significant impact and may markedly improve the total process yield and the product quality and enhance the economy of the entire process [11]. Meeting this challenge can be enhanced by incorporating in-line monitoring for acquiring critical information on process parameters and attributes [12]. The ideal in-line monitoring technique should fulfil certain requirements, including high sensitivity, rapid response time, high accuracy and robustness. Moreover, it should cover a wide dynamic range, a low limit of detection, have minimal recalibration needs and comply with Good Manufacturing Practice (GMP). However, thus far, in-line monitoring opportunities are sparsely discussed in downstream processing.

During the virus production process, metabolites, nutrients and HCP are either consumed or released into the medium. To follow the production process, these substances are monitored. At the end of the virus production process, the medium collected at harvest contains, in addition to the vaccine virus, all of these substances. A DSP process that eliminates process- and product-related impurities is expected to remove those substances. The chemical analyzer Cobas integra 400 plus has many advantages that make it a suitable candidate for an in-line monitoring technique, including the following: 1. It has a rapid response time (can run up to 400 tests per hour, the reagents are ready-to-use and each pack holds all the necessary reagents for up to 800 determinations). 2. It has minimal recalibration needs (the reagents have long stability requiring minimal calibration efforts. The samples and reagents are refrigerated, preventing reagent evaporation and degradation and ensuring long-term on-board stability and long inter-calibration intervals). 3. It covers a wide dynamic range. 4. It is highly accurate and robust (the format of the reagents standardizes results across integrated laboratory networks and can be automatically handled by the system, reducing the possibility of errors and saving staff time). 5. It is very handy (each assay uses 2- to 10-microliter samples and single-use cuvettes to ensure carryover free analytics).

Herein, a DSP in-line monitoring approach that is based on following the concentration of metabolites, nutrients and an HCP representative using a chemistry analyzer is proposed. The analysis of samples from different purification steps using the chemical analyzer revealed critical information on the purification process’ efficiency. The method is rapid, efficient, sensitive and accurate. The integration of a chemical analyzer in process development and pharmaceutical manufacturing processes can be a useful tool for effectively obtaining adequate in-line process data and achieving improved process effectiveness. The adoption of this technique during the purification process can improve the process yield and the product quality and enhance the economy of the entire downstream process for biotechnology and bio pharmaceutical products.

## 2. Materials and Methods

### 2.1. Virus Upstream Production

The rVSV-∆G-spike vaccine was produced in BioBLU^®^ 5p Eppendorf single-use bioreactors (Hamburg, Germany) [6] or in multi trays (MT) (NUNC EasyFill Cell Factory System, Thermo Fisher Scientific, Waltham, MA, USA). Vero cells were absorbed to fibra-cel within the bioreactor/MT and grown for six days. Cells were then infected with the vaccine virus, which was allowed to replicate in the cells for 48 h. Infection was carried out at a multiplicity of infection (MOI) of 0.1 (The MOI refers to the number of virions that are added per cell during an infection; an MOI of 0.1 means that 1 virion is added per 10 cells). The medium from the bioreactor/MT, containing the released vaccine virus, was harvested into a Flexboy bag (Sartorius, Gottingen, Germany). The bag was transferred to the downstream process.

### 2.2. Virus Purification

**Figure 1:**
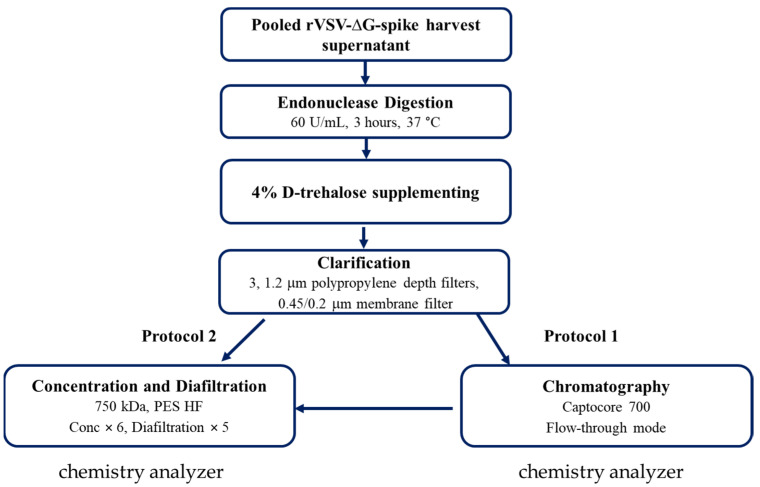
A scheme of the different rVSV-∆G-spike purification DSP steps showing the two different purification protocols and the steps that were analyzed with the chemistry analyzer.

The DSP of rVSV-∆G-spike comprises the several steps described in [9,10]. Briefly, MT or bioreactor harvested supernatants were subjected to endonuclease digestion of host cell DNA using 60 U/mL Denerase endonuclease (20804-5M, GMP grade, c-LEcta, Leipzig, Germany) in the presence of 2 mM MgCl_2_ (Spectrum chemical, New Brunswick, NJ, USA) for 3 h in a shaker incubator (Innova 43R, Eppendorf, Hamburg, Germany) at 37 °C under constant and mild agitation. The endonuclease-digested cell culture harvested supernatants were supplemented with 4% D-trehalose (Pfanstiehl, Waukegan, IL, USA) and then clarified using a filter train with pores of 3 µm (sartopure PP3 size 8, 0.12 m^2^, polypropylene, Sartorius, Goettingen, Germany), 1.2 µm (sartopure PP3 size 8, 0.09 m^2^, polypropylene, Sartorius, Goettingen, Germany) and 0.45/0.2 µm (sartopore platinum, size 8, 0.13 m^2^, PES, Sartorius, Goettingen, Germany). After endonuclease digestion, the following two different purification protocols were carried out: with chromatographic purification (protocol 1) or without chromatographic purification (protocol 2). In protocol 1, the next step was chromatographic purification (flow-through mode), with AKTA Pilot 600 equipped with captocore 700 resin packed in Index 100/50 column (Cytiva, MA, USA). The last step, in both protocols, was tangential flow filtration (TFF) using the uniflux 10 TFF system equipped with polysulfone (PS) 750 kDa molecular weight cutoff, a 1-millimeter internal diameter hollow fiber filter (Cytiva, MA, USA). The virus-containing solution was concentrated 6-fold by volume and diafiltered with five diafiltration volumes against an equilibration buffer (20 mM tris-HCl, 100 mM NaCl, 4% D-trehalose, pH = 7.2). A scheme of the different rVSV-∆G-spike DSP purification steps including the two different protocols (1 and 2) and the steps that were analyzed with the chemistry analyzer are presented in Figure 1.

### 2.3. Chemistry Analyzer: Cobas Integra 400 Plus

The Cobas Integra 400^®^ Plus (Roche Diagnostics GmbH, Mannheim, Germany) is an automated wet chemistry analyzer with comprehensive testing capabilities. A few tens of samples, taken from different steps of the DSP from different protocols, were tested with the Cobas chemistry analyzer. A total of 100 µL of each sample was placed into Eppendorf tubes (with no cap) and tested for GlutaMAX (AQB), lactate (LAC2B), glucose (GLC2B), ammonia (NH3B), glutamate (GLU2B) and LDH using commercially available kits from Roche. The concentration of each sample after harvest was set to 100% and the other results were normalized accordingly. Means and SDs were calculated from 3 different DSP processes.

### 2.4. Host Cell Protein (HCP) Analysis

Residual proteins of Vero cells were measured using a Vero cell host cell protein (HCP) ELISA kit (Cygnus Technologies, Southport, NC, USA), following the manufacturer’s instructions. A total amount of 50 µL of diluted samples were added to microtiter strips coated with an affinity purified capture polyclonal goat anti-Vero cell antibody. Horseradish peroxidase (HRP) enzyme labeled anti-Vero cell antibody (goat polyclonal, 100 µL) was added to wells and incubated for 2 h at 25 °C with shaking at 500 rpm. The wells were washed 4 times with wash buffer to remove unbound reactants. An amount of 100 µL of 3,3′,5,5′ tetramethylbenzidine (TMB) solution was immediately added to the plate and incubated at 25 °C for 30 min. The reaction was stopped using 100 µL of 0.5 M H_2_SO_4_ stop solution and the absorbance was read at 450 nm using a CLARIOstar multi-mode reader (BMG LABTECH, Ortenberg, Germany). All standards, controls and samples were assayed in duplicate. The residual Vero HCP concentration (ng/mL) in a tested sample was calculated by interpolation from a 4-parameter non-linear fit regression of the standard curve after subtracting the blank.

## 3. Results and Discussion

**Figure 2:**
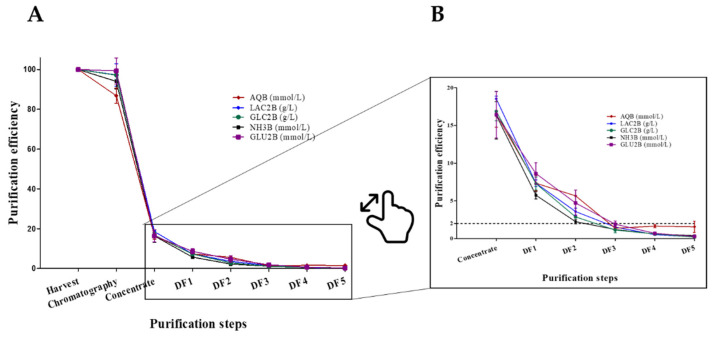
Metabolite and nutrient purification efficiencies for three DSPs. Each metabolite/nutrient was measured in each step of the DSP. The initial concentration was set as 100% and all the others were normalized accordingly. The results from three processes were averaged and the SD was calculated (**A**). Zoom in to purification efficiencies of the diafiltration steps (**B**). The dashed line represents 2% of the initial concentration.

This study was mainly focused on the application of a chemical analyzer to improve the process development and manufacturing processes. Chemical analyzer integration in the DSP enabled in-line monitoring of contaminant removal and, therefore, inspection and control of purification progress. The concentration of several metabolites and nutrients in all the steps of the DSP was analyzed using a chemical analyzer. The analysis included the following: 1. GlutaMAX (Ala-Gln di-peptide, AQB), which replaced Glutamine (Gln) in the medium, since the latter is unstable and its oxidization releases ammonia (NH_3_), which is toxic to cells. 2. Lactate (LAC2B), which is produced by cells in the process of using an energy source (such as glucose). 3. Glucose (GLC2B), which is an energy source. 4. Ammonia (NH3B), which is usually produced as a result of glutamine disassembly and is toxic to cells. 5. Glutamate (GLU2B), which is the oxidation product of glutamine and has a central role in the metabolism. The concentration values from all the DSP steps were normalized by dividing the value for a specific step by the value of the harvest. Since the initial concentration of each metabolite/nutrient differed between the harvests, all the initial/harvest concentrations were set to 100%. The normalized results taken from three different DSPs are shown in Figure 2A. Two of the DSPs were conducted on a harvest obtained after growing cells in multi-trays and the third harvest was from bioreactors. The reduction from a 100% harvest concentration is shown for the following DSP steps: Chromatography, Concentrate and Diafiltration (DF 1–5).

In protocol one (Figure 1), chromatography process was performed with captocore 700 resin. This resin contains 700 kDa diameter pores that are only positively charged (strong anionic exchanger) inside the pores. Therefore, only anionic molecules that are <700 kDa (and >2 kDa) are bound to the resin, while all the others will move with the flow-through. As indicated in Figure 2A, after chromatography, most of the metabolites/nutrients remained at the same concentration and were not removed in the chromatography step. As expected, small cationic and uncharged molecules (such as NH_3_ and glucose) were not bound to the anionic exchange resin. In addition, the anionic small molecules (such as glutamate and lactate) did not bind to the resin (despite their charge) because of their small size. On the other hand, a small reduction (~15%) in the AQB (Ala-Gln di-peptide) concentration was noted, though this molecule is small and uncharged. This small reduction may result from the non-specific absorption of this peptide due to hydrophobic interactions.

The next DSP step was Tangential Flow Filtration (TFF), which consists of concentration and buffer exchange (diafiltration) [13]. This is an important step in the DSP of the rVSV-∆G-spike to reduce the viral harvested volume while increasing the viral concentration, achieve solution exchanges and reduce the impurity load for the subsequent purification stages. The TFF step uses a 750 kDa hollow fiber and includes five cycles of diafiltration (DF) against a dialysis buffer with similar pH and conductivity values as the chromatography (protocol one) or the harvest (protocol two) products. The similar values (pH, conductivity, etc.) of the chromatography and the dialysis buffer make the determination of complete dialysis difficult based on the common parameters such as pH and conductivity. Therefore, for this process, the chemistry analyzer was used to determine the dialysis progress, discover the point of complete dialysis and evaluate the vaccine substance purification progress from contaminating metabolites and nutrients during TFF. Based on Figure 2A, the following two points should be noted and elaborated: 1. The similarities between metabolites/nutrients purification efficiencies profiles, and 2. the resemblance between different DSPs.

The TFF process began with a six-fold concentration step (from 12 to 2 L). For all the metabolites tested, a large drop in concentration (80%) appeared in this step (despite the concentration). In addition, low SD values were observed, despite all the differences between the biological processes (cell density, cell metabolism, proliferation, virus production), and the different sources of the DSP. In the first diafiltration cycle (DF1), 90–95% of the small molecules were removed. In the second cycle (DF2), 95–97%, and in the third cycle (DF3) 98–99% of the small molecules were eliminated. In the fourth and fifth cycles (DF4 and DF5), the concentrations of small molecules were below the detection threshold (Figure 2B). The TFF process using a 750 kDa hollow fiber filter efficiently removed the small molecules. Therefore, it was concluded that three diafiltration cycles are sufficient to remove the majority of small contaminating molecules from the vaccine substance. It was, therefore, decided to stop the diafiltration after three cycles. These results clearly demonstrate the utility of the chemistry analyzer for monitoring the removal efficiencies of small contaminating molecules, such as metabolites and nutrients, from the pharmaceutical substance. This method enables a diafiltration cycle number to be determined, which is needed for the efficient purification of the vaccine substance at the TFF stage.

Another substance that was analyzed is Lactate dehydrogenase (LDH), a soluble cytosolic enzyme presents in most eukaryotic cells that is released into culture medium upon cell death due to damage to the plasma membrane. LDH is an enzyme of the dehydrogenase family that functions as a catalyst for the redox reaction of lactate and pyruvate. LDH is defined as a contaminant protein in viruses and recombinant protein drug substances that are produced from cell cultures. In practice, LDH is one of the main HCPs that are produced in the cell culture fermentations [14]. Tracking LDH removal in different steps of the DSP can help in selecting desired DSP steps and their order of performance. The HCP proteins are defined as contaminating proteins that should be removed from the vaccine substrate during the purification process; hence, LDH removal serves as a convenient indicator of HCP removal.

**Figure 3:**
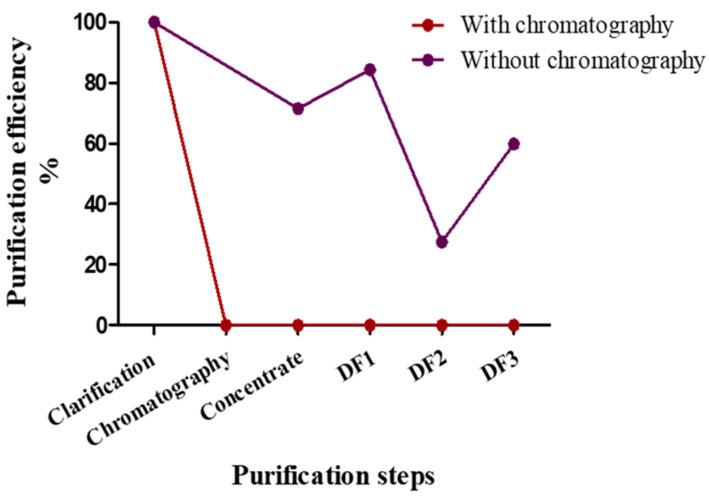
LDH purification profiles in protocol 1 (with chromatography, red line) and protocol 2 (without chromatography, purple line). LDH was measured in each step of the DSP. The initial concentration was set as 100% and all the others were normalized accordingly.

An analysis of the LDH concentration was performed with the chemistry analyzer for the two different DSPs (protocol one and two). As can be seen in Figure 3, the LDH was efficiently removed in the chromatography stage using the captocore 700 strong anion exchanger (red line). Since LDH is a 140 kDa anion, its complete removal by the chromatography is expected. In contrast, the TFF only removed the LDH enzyme partially (40–60%), although the LDH molecular weight is much smaller than the TFF cutoff (purple line). This can be explained by the high hydrophobicity of the LDH molecule and its possible binding to other hydrophobic molecules in the vaccine substance, that results in aggregation and, hence, the inability of the TFF to remove LDH from the solution. Since LDH is one of the main HCPs, the purification profiles of LDH and the total HCP in the same DSPs were compared. The analysis of the total HCP was conducted using an ELISA. A similar pattern was found for LDH and total HCP removal (Table 1). The chromatography by captocore 700 decreased the HCP concentration to almost zero (from 100 to 0.6%), whereas TFF resulted in a ~40–50% clearance. These results demonstrate the essentiality of the chromatography process for the complete DSP. In conclusion, these results validate the application of concentration analysis using a chemistry analyzer for the in-line monitoring of DSP efficiency.

## 4. Conclusions

In summary, we propose employing an in-line monitoring technique in downstream purification processes. The application of this technique was demonstrated in the DSP for a VSV-based SARS-CoV-2 vaccine. Sample analysis from all the purification steps with the chemistry analyzer revealed critical information on process parameters and attributes. All the small molecule metabolites were removed in the diafiltration steps performed by only three diafiltration cycles. The removal of more than 98% of the metabolites after three diafiltration cycles is critical in meeting the guidelines of the regulatory authorities. Having only three cycles (and not five as initially thought) lowers the duration and expense of the DSP. The reduction in DF cycles also improves the process yields since the virus is sensitive to the continuous shear forces that occur during the TFF process. Moreover, the same pH and conductivity of the dialysis buffer and the chromatography buffer made it difficult to follow the diafiltration outcome. Sample analysis with the chemistry analyzer overcame these limitations. The analysis also showed that LDH was removed completely using chromatography. Altogether, analyzing samples with the chemistry analyzer is ideal as an in-line monitoring technique: It is rapid, efficient, sensitive, accurate and requires only minimal recalibrations. The adoption of this technique during the purification process can improve the process yield and the product quality and enhance the economy of the entire downstream process for biotechnology and bio pharmaceutical products.

## Figures and Tables

**Table 1:** HCP purification efficiencies in the DSP with and without chromatography.

	Purification Step	Purification Efficiency %
With chromatography	clarification	100
	Chromatography	0.6
	DF1	0.2
	DF3	0.2
Without chromatography	clarification	100
	DF1	52
	DF3	58

## Data Availability

The data that support the findings of this study are available from the corresponding author upon reasonable request.
